# Preparation of xyloglucan-grafted poly(N-hydroxyethyl acrylamide) copolymer by free-radical polymerization for in vitro evaluation of human dermal fibroblasts

**DOI:** 10.1007/s10856-024-06783-1

**Published:** 2024-03-25

**Authors:** Maykel González-Torres, Ricardo Martínez-Mata, Erika Karina Ruvalcaba-Paredes, Alicia del Real, Gerardo Leyva-Gómez, Alfredo Maciel-Cerda

**Affiliations:** 1 CONAHCYT & Laboratorio de Biotecnología, Instituto Nacional de Rehabilitación “Luís Guillermo Ibarra,”, Ciudad de Mexico, 14389 Mexico; 2 Instituto de Investigaciones en Materiales, Universidad Nacional Autónoma de México, 04510 Mexico DF, Mexico; 3 Centro de Física Aplica y Tecnología Avanzada, Universidad Nacional Autónoma de México, Boulevard Juriquilla 3001, Querétaro, 76230 Mexico; 4 Departamento de Farmacia, Facultad de Química, Universidad Nacional Autónoma de México, Ciudad de Mexico, 04510 Mexico

## Abstract

Xyloglucan is a rigid polysaccharide that belongs to the carbohydrate family. This hemicellulose compound has been widely used in biomedical research because of its pseudoplastic, mucoadhesive, mucomimetic, and biocompatibility properties. Xyloglucan is a polyose with no amino groups in its structure, which also limits its range of applications. It is still unknown whether grafting hydrophilic monomers onto xyloglucan can produce derivatives that overcome these shortcomings. This work aimed to prepare the first copolymers in which N-hydroxyethyl acrylamide is grafted onto tamarind xyloglucan by free-radical polymerization. The biocompatibility of these structures in vitro was evaluated using human dermal fibroblasts. Gamma radiation-induced graft polymerization was employed as an initiator by varying the radiation dose from 5–25 kGy. The structure of the graft copolymer, Xy-g-poly(N-hydroxyethyl acrylamide), was verified by thermal analysis, Fourier transform infrared spectroscopy, and nuclear magnetic resonance spectroscopy. The findings indicate that the degree of grafting and the cytotoxicity/viability of the xyloglucan-based copolymer were independent of dose. Notably, the grafted galactoxyloglucan exhibited efficient support for human dermal fibroblasts, showing heightened proliferative capacity and superior migration capabilities compared to the unmodified polymer. This copolymer might have the potential to be used in skin tissue engineering.

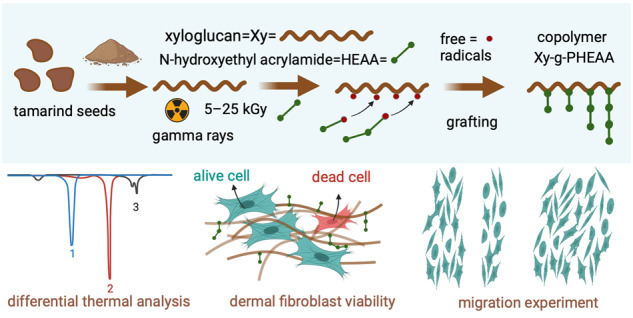

## Introduction

Polysaccharides (PSs) are carbohydrate macromolecules that originate from animal and plant sources and are abundant in nature [1]. Due to their high availability, noncarcinogenicity, and biocompatibility, there is considerable interest in using them as renewable sources to manufacture functional materials [2]. Among PSs, xyloglucan (Xy), a hemicellulose compound, has advantages of biodegradability, nontoxicity, and low cost to produce biomaterials with good biointerface performance [3]. In addition, galactoxyloglucan’s mucoadhesive, pseudoplastic, and mucomimetic properties make this compound suitable for drug delivery systems [4]. However, two drawbacks of Xy are the rigidity of the molecule and a lack of amino groups in the structure. The absence of amino groups in the native Xy might limit its direct reactivity or interaction with certain functional moieties. Amino groups are commonly involved in bioconjugation reactions, such as coupling with reactive species for grafting or linking to other polymers or biomolecules. Therefore, chemical modification is necessary to overcome these shortcomings and expand the application fields of this polyose in biotechnology [5].

Grafting is among the most promising chemical modification methods for Xy [6]. In grafting, a monomer or polymer is covalently attached to the PS, which allows the synthesis of various hybrid biopolymers or multicomponent systems of practical usefulness in biomedical applications [7]. Several works have used graft polymerization to improve the chemical properties of Xy using various initiation methods or direct modification of PSs [8]. Octenyl succinic anhydride (OSA) was used to generate Xy-OSA amphiphilic molecules with potential application in drug delivery [9]. The biocompatibility of Xy has been improved by the grafting of polylactic acid (PLA) via ring-opening polymerization (ROP), which has allowed the preparation of composite materials in the form of reinforced fibers [10]. Grafting reactions with redox initiators have attracted increasing scientific interest because of their simplicity and minimal side reactions. Tamarind Xy has been grafted with acrylamide (AAm) and polyacrylamide (PAAm) using ceric ammonium nitrate (CAN) [11, 12]. Ammonium persulfate (APS) has been used to graft polyacrylic acid (PAAc)/diatomite (D) to create a superabsorbent Xy-g-PAAc/D [13]. Sorbents generated from Xy grafted via redox initiation, such as poly(2-hydroxyethyl methacrylate) (pHEMA) grafting, have applications in removing vanadium (V) ions from aqueous solutions, which helps prevent carcinogenic diseases derived from the ingestion of this metal above the acceptable dose levels [14].

Furthermore, microwave-initiated grafting has been widely used to assist redox synthesis [6]. PAAm has been grafted onto tamarind Xy by microwave-assisted polymerization, and the generated derivative (Xy-g-PAAm) has been used for nanofabricating wound dressings [15]. In addition, mucoadhesive polymers (Xy-g-poly(n-vinyl pyrrolidone)) [16] and flocculating agents (Xy-g-polyacrylonitrile) have also been prepared by the above-described method [17].

Moreover, atom transfer radical polymerization (ATRP) has been demonstrated experimentally to achieve the grafting of poly(methyl methacrylate) (PMMA) for use in removing toxic dyes [18]. Our group has previously grafted ethyl acrylate onto tamarind xyloglucans using free radicals to generate biodegradable films [19]. To our knowledge, gamma radiation has yet to be used to create copolymers of these polysaccharides.

This work aims to synthesize and characterize copolymers based on N-hydroxyethyl acrylamide (HEAA) grafted onto the Xy of tamarind by radical polymerization and to determine whether these structures have characteristics that allow their future application in skin tissue engineering (STE).

The radiation-induced graft polymerization method was used in this work. The copolymers (Xy-g-PHEAA) were characterized by solid-state carbon-13 nuclear magnetic resonance (^13^C-CP/MAS NMR), differential scanning calorimetry (DSC), Fourier transform infrared (FTIR) spectroscopy, and thermogravimetric analysis (TGA). The in vitro biocompatibility of Xy-g-PHEAA was evaluated by 3-(4,5-dimethylthiazol-2-yl)-2,5-diphenyltetrazolium bromide (MTT) cytotoxicity, viability (calcein/ethidium homodimer assay), and proliferation (KI67 expression by immunofluorescence) studies involving human dermal fibroblasts (HDFs).

The monomer HEAA has been used to generate superhydrophilic antifogging coatings in engineering applications [20]. Grafting of HEAA has been shown to increase the antibacterial efficacy of the generated derivatives, their resistance to bacterial adhesion, and their bacterial killing activity [21]. The hydrophilic properties and the presence of an amino group that allows the formation of semi-interpenetrated polymeric networks make this monomer a suitable candidate for modifying Xy obtained from tamarind seed.

The use of Xy for STE has yet to be well studied. The application of Xy in treating corneal epithelial lesions has been reported [22]. Previous research suggested that Xy could promote human skin regeneration by influencing fibroblast proliferation and migration [23]. The successful use of Xy as an extracellular matrix for hepatocyte culturing has also improved the biocompatibility of these biomaterials [24]. In addition, polyvinyl alcohol grafting onto Xy to prepare hydrogels for skin wound dressings has been studied [25]. Therefore, in this work, it is hypothesized that it is possible to graft HEAA onto Xy using gamma radiation-induced graft polymerization to improve the hydrophilicity and decrease the rigidity of the starting PS. The copolymers obtained could have potential applications in skin bioengineering.

The significance of this study, along with the innovative xyloglucan grafted with HEAA, resides in unveiling the potential of Xy. This research positions Xy as a valuable asset for biomedical applications. The grafted Xy emerges as a versatile and functional copolymer, showcasing augmented thermal stability, heightened proliferative capacity, and superior migration capabilities when contrasted with the pristine polysaccharide.

## Materials and methods

### Chemicals

Tamarind seeds, from which Xy is obtained, were obtained from industrial waste in Mexico. The seeds were manually separated from the pulp and briefly soaked in hot water (90 °C). The testa was removed, and the seeds were first sun-dried and later dried in an oven (Scorpion Scientific A50980) at 35 °C for at least 5 h for each procedure. Then, the seeds were milled, and a #80 mesh sieve was used to separate the ground seeds by following ASAE standards [26]. The tamarind kernel powder was treated with ethanol to obtain the Xy. N-Hydroxyethyl acrylamide (HEAA, 7646-67-5), ethanol (purity > 99.45%, CAS-No.: 64-17-5), and acetone (purity > 99.5%, CAS-No.: 67-64-1) were purchased from Merck (Germany). The solvents were used as received, while HEAA was distilled to eliminate the inhibitor.

### Preparation of Xy-g-PHEAA

HEAA was grafted onto xyloglucan Xy by gamma radiation-induced graft polymerization using 400 mg of Xy, 2.5 ml of ethanol, and 500 microliters of monomer. Doses from 5 to 25 kGy in increments of 5 kGy (D1, D2, D3, D4, and D5) were used. A Transelektro-LGI-01 ^60^Co-gamma-ray source was used to irradiate the substances in Pyrex tubes (1.52 kGy/h). An Amber 3042 dosimeter (Perspex Harwell) was used to measure the doses accurately. For easy classification of the grafted Xy, the resulting copolymers were denoted according to the dose, namely, XyM2D1, XyM2D2, XyM2D3, XyM2D4, and XyM2D5, where M2 represents HEAA. Soxhlet extraction with acetone (250 ml) was used to purify the Xy-g-PHEAA from HEAA and PHEAA residues for approximately 72 h.

### Characterization methods

#### Degree of grafting

The following equation was used to calculate the degree of grafting (W_Xy-g-PHEAA_) of PHEAA onto Xy:

$${W}_{Xy-g-PHEAA}=\frac{(\,{m}_{Xy-g-PHEAA}-{m}_{Xy})}{{m}_{Xy}}\times 100$$

${m}_{Xy-g-PHEAA}$ was determined after the grafting and purification processes, and ${m}_{Xy}$ is the initial mass of tamarind Xy.

#### Nuclear magnetic resonance

Nuclear magnetic resonance (NMR) spectra of Xy and Xy-grafted PHEAA were obtained on a Bruker Avance 400 spectrometer (400 MHz) with a high-resolution magic angle spinning (HR-MAS) probe. The PS samples were first swollen in D_2_O, placed on a 4 mm zirconia rotor, and rotated at 5 kHz. ^1^H and ^13^C NMR spectra were recorded using tetramethyl silane (TMS) as a reference.

#### Infrared spectroscopy

Fourier transform infrared (FTIR) spectra of pristine (Xy) and Xy-g-PHEAA samples were obtained by means of an attenuated total reflectance (ATR) FTIR Bruker vector spectrometer equipped with a ZnSe ATR accessory. The powders were scanned from 4000 to 600 cm^−1^ at 4 cm^−1^ resolution. The samples were dried under vacuum before the analysis.

#### Thermal analysis

Thermal analysis (TA) of the chemical changes of Xy as a function of temperature was performed by thermogravimetry (TGA) using a thermoanalyzer (Q5000IR, TA instrument). The energy required by the starting and Xy-based polymers to increase the sample temperature was monitored by modulated differential scanning calorimetry (MDSC Q100, TA instrument). A 10 °C/min heating rate and a nitrogen atmosphere (N_2_) were used for the thermal analysis with a gas flow of 25 ml/min. The temperature range for TGA was 27–600 °C, while MDSC required a range from −20 to 300 °C. Aluminum crucibles were used to contain 5 mg of the polysaccharides.

### Cellular studies

#### Cytotoxicity

An MTT assay was carried out to determine the cytotoxicity of neat Xy and the synthesized (Xy-g-PHEAA) samples. Five milligrams of the control or modified hemicellulose compound was added to each well, and dermal fibroblasts were plated at a density of 10^3^ cells per well. In this experiment, the reduction of 3-(4,5-dimethylthiazol-2-yl)-2,5-diphenyltetrazolium bromide to formazan was measured with a microplate reader (Synergy™ HTX Multimode reader, USA) at 540 nm. Cell survival below 70% was considered cytotoxic (ISO 10993-5:2009).

#### Viability

The viability of HDFs cocultured with Xy or Xy-g-PHEAA was examined by a Live/Dead™ kit. By following the Calcein/Ethidium Homodimer I dye (EthD-1) kit’s guidelines (Thermo), 2 μM ethidium homodimer and 1 μM calcein diluted in Dulbecco’s modified Eagle medium (DMEM) were used and incubated for 1 h. Briefly, the cells were visualized with a Carl Zeiss confocal microscope (LSM 780). Viable HDFs were appeared green, while nonviable cells appeared red.

#### Proliferation and immunophenotype assays

Immunofluorescence (IF) is a molecular technique used in conjunction with fluorescence microscopy for light microscopy and is primarily used for microbiological samples. This technique exploits the specificity of antibodies to antigens to direct fluorescent dyes to specific biomolecular targets within cells, thereby visualizing the distribution of target molecules in a sample. Here, the anti-Ki67 antibody (ab16667, Abcam, UK) was coupled to AF488 (A11008, 1:500, Invitrogen) to visualize the nuclear protein (ki67) expression associated with cell proliferation.

Additionally, anti-fibroblast surface protein [ab11333, 1B10, 1:100, Abcam, UK] was mixed with AF594 (secondary antibody, Catalog # A11005, 1:500, Invitrogen, USA) to determine whether the HDF lineage remained unchanged after 24 and 48 h of culture for the control and Xy-based copolymer with PHEAA.

#### Migration assay

HDFs were cultivated in 12-well plates, with a seeding density of 2.0 × 10^6 cells per well and nurtured at 37°C with 5% CO_2_ until confluency was achieved. To impede cell proliferation, the medium underwent substitution with mitomycin-containing serum-free DMEM for a 4-h period. A uniform monolayer was then meticulously disrupted through a scratch using a sterile 1 mL pipette tip. Post-scratching, the culture milieu experienced renewal with fresh serum-free DMEM. Thereafter, an Axio Observer A1 (Carl Zeiss), featuring an LED light source Colibri and a Carl Zeiss digital camera, captured images (*n* = 10 per treatment) from the well centers at time points 0, 24, 48, and 72 h post-scratching.

### Statistical analysis

Origin 9.0 software (Origin Lab, USA) was used for plotting the results of spectroscopic and thermal analyses. Conversely, GraphPad Prism (GraphPad Software, USA) was used to plot the results of biological experiments. Within a group of values (*n* = 10), statistical significance (*) was defined for outcomes with *P* values less than 0.05. One-way analysis of variance (ANOVA) was used for viability experiments (*n* = 10, α = 0.05).

## Results and discussion

### The synthesis, proposed mechanism, and degree of grafting of Xy-g-PHEAA

**Fig. 1:**
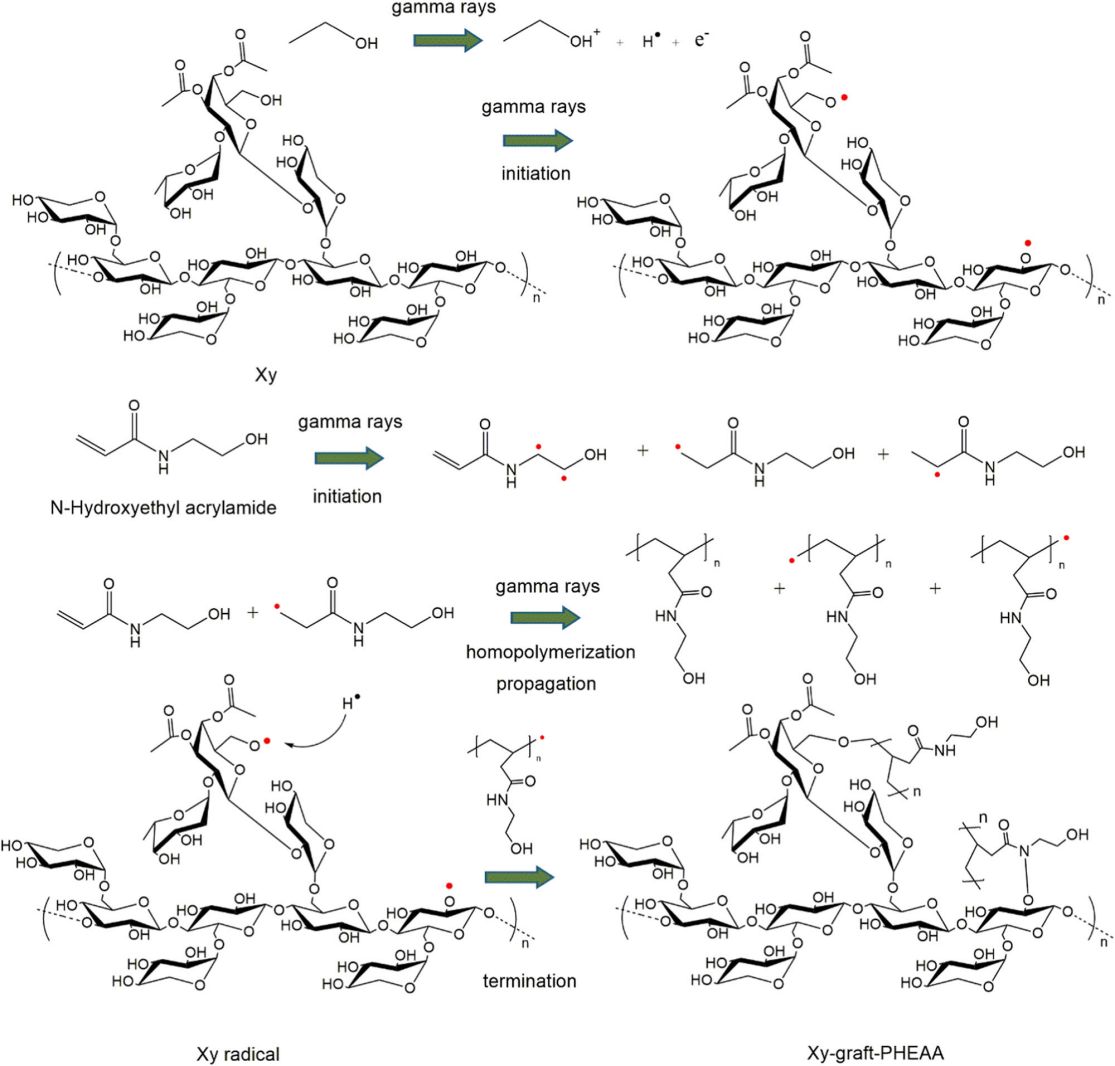
Schematic of Xy and Xy-graft HEAA copolymers prepared via the simultaneous irradiation method

Figure 1 shows a schematic of the preparation of Xy-g-PHEAA based on radiation-induced grafting of PHEAA onto Xy. First, primary ethanol radicals are produced. The incidence of gamma rays also yields Xy and HEAA primary radicals in the initiation step. The Xy primary radicals are necessarily distributed in the alcohol groups by the scission of hydrogen (Xy•). HEAA’s primary radical can be positioned at any α, β, γ, or δ carbon of the monomer with similar opportunities to react (HEAA•). HEAA precursors can initiate polymerization by reacting with the monomer to yield PHEAA macroradicals (PHEAA•). In the last step, Xy• may be deactivated by recombination with any radical in the graft reaction. The deactivation of the PS by the homopolymer’s growing radical chain can form the Xy-grafted PHEAA copolymer.

On the other hand, the degree of grafting (${W}_{Xy-g-PHEAA}$) ranged from 82 to 94%, with no significant difference between the groups, indicating that it is independent of the dose (Fig. S1). Some earlier works on Xy-g-PAAc did not show data to compare the degree of grafting [13]. The degree of grafting for Xy-g-AAm (45.6% on average), initiated by ceric ions and prepared from tamarind mucilage, was lower than that of Xy-g-PHEAA [11]. Conversely, when tamarind seed was used with ceric ammonium nitrate as the initiator, the degree of grafting increased to more than 140% and reached a maximum of 667.8% [27]. The same degree of grafting (80% on average) was observed for Xy-grafted poly(ethyl acrylate) initiated by gamma radiation [19]. Therefore, the degree of grafting was the first evidence of the grafting of PHEAA onto Xy. However, our work was limited to one type of gravimetric measurement, while others have reported the grafting efficiency and conversion parameters to enhance understanding of the synthesis [15].

### Structure and properties of Xy-g-PHEAA

#### Searching for evidence of grafting using NMR

**Fig. 2:**
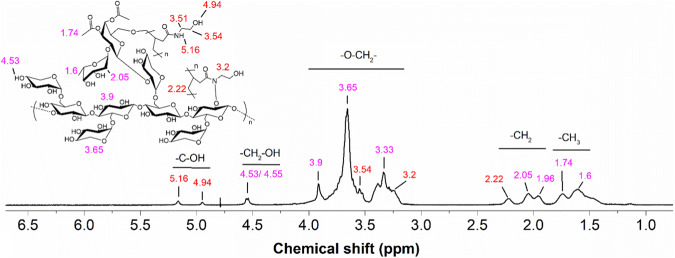
NMR of Xy-g-PHEAA

The ^1^H-NMR spectrum of the Xy-grafted PHEAA copolymer is shown in Fig. 2. The signals at 4.53/4.55 ppm (–CH_2_–OH), 3.90/3.50/3.33 ppm (–O–CH_2_), 2.05/1.96 ppm (–CH_2_), and 1.74/1.60 ppm (–CH_3_), which are highlighted in purple, were attributed to Xy. This signal assignment was based on the previous work reported for the copolymerization of poly(ethyl acrylate) onto tamarind kernel powder (Fig. S2) [19]. New signals were observed at 5.16/4.94 ppm (–C-OH), 3.54/3.20 ppm (–O–CH_2_), and 2.22 ppm (–CH_2_), which confirmed the grafting of PHEAA. The signal typically observed at 5.27 ppm for Xy shifted in the high field direction to 4.53/4.55 ppm for Xy-g-PHEAA. The integrations for the copolymer signals were 2H (–NH), 1H (–C–OH), 1H (–O–CH_2_), and 3H (–CH_2_). The peaks in the spectrum attributed to the grafting positions and the attached molecule were identified in red for improved clarity.

The above-described ^1^H NMR control signals aligned with those reported for Xy extracted from apple pomace [28]. The positions of the PHEAA peaks were compared to those of the poly(2-(methacryloyloxy) ethyl trimethyl ammonium-g-N-hydroxyethyl acrylamide) nanogels and PHEAA-grafted polystyrene (PSt), with substantial similarity in the –C-OH, –O-CH_2_, and -CH_2_ proton signals [29, 30].

#### Searching for evidence of grafting using FTIR

**Fig. 3:**
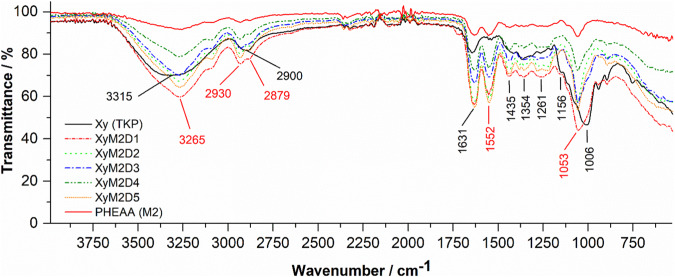
FTIR spectra of Xy and Xy-g-PHEAA

The FTIR spectra of the starting polymer (Xy or TKP), the grafted polymer (PHEAA or M2), and the copolymer (Xy-g-PHEEA) are shown in Fig. 3. The spectra included the dose effect for the graft copolymer. First, the Xy spectrum showed bands at 3315 cm^−1^, 2900 cm^−1^, 1631 cm^−1^, and 1006 cm^−1^, which were ascribed to hydroxyl (–OH) stretching, –CH asymmetric stretching, -OH bending vibrations, and –CO stretching frequencies [13]. The bands observed at 1156–1435 cm^−1^ were attributed to –CH angular deformations. The characteristic absorption peaks at 1645 cm^−1^, 1549 cm^−1^, and 1067 cm^−1^ were attributed to the –C = O group (amide I), –NH bending vibrations (amide II), and –CO/–CN stretching of PHEAA [31]. The peaks in the Xy-g-PHEEA spectra provided the first experimental evidence that PHEAA grafting did not depend on the dose. The –OH stretching band shifted to a higher wavenumber (3265 cm^−1^), suggesting a new type of interaction. The –CH asymmetric stretching gave rise to two bands (2930 and 2879 cm^−1^), suggesting novel vibrational interactions for grafted Xy. The more striking grafting evidence was the appearance of a band at 1552 cm^-1^, which was clearly attributed to grafted PHEAA –NH bending [32]. Finally, the peaks attributed to –CO stretching shifted to a lower wavenumber (1053 cm^-1^), consistent with the above findings. As illustrated in Fig. 3, the spectral comparison of the pristine and treated samples confirmed that PHEAA was grafted onto Xy, affecting the hydrogen bonding formation (broader –OH stretching bands) and –OH vibrational spectroscopy. Due to its enhanced intermolecular forces, this grafted structure may be helpful as a biomimetic material for tissue adhesion functions and STE [33].

#### Study of the Xy and Xy-g-PHEAA thermodynamic properties

**Fig. 4:**
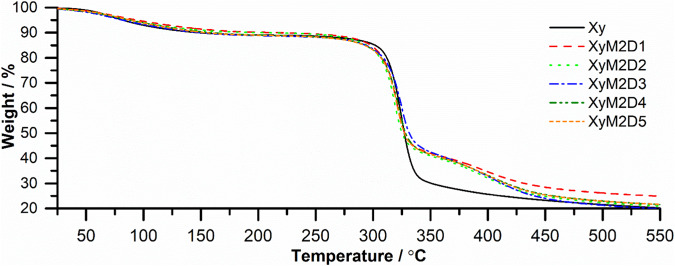
TGA of Xy and Xy-g-PHEAA copolymers synthesized at different doses

**Fig. 5:**
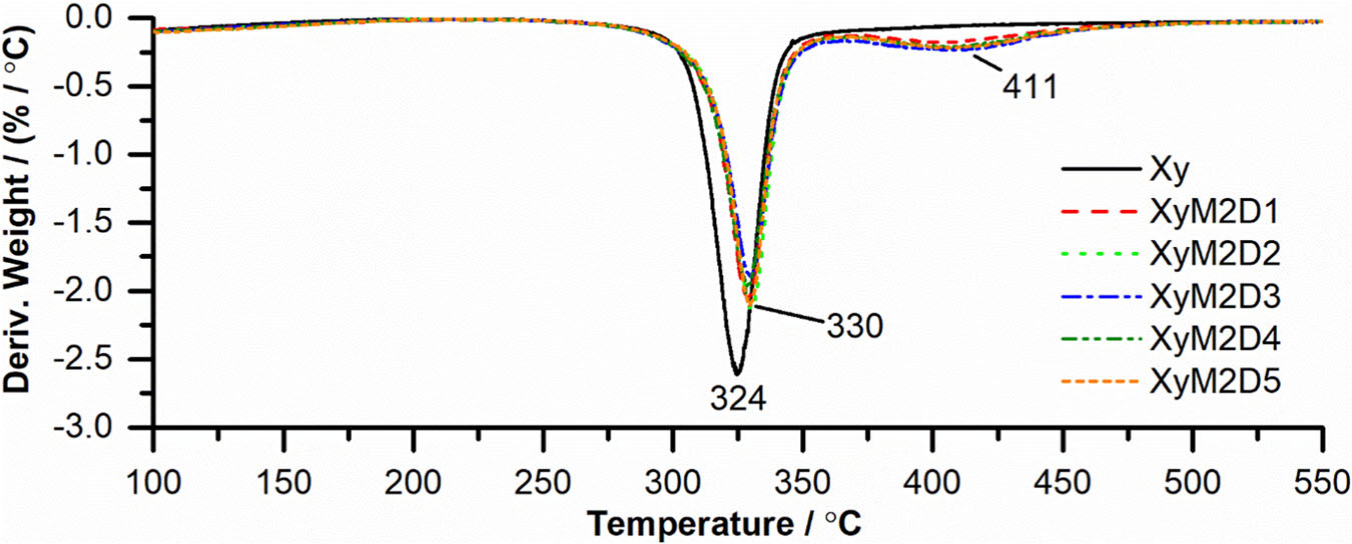
DTGA of Xy and Xy-g-PHEAA copolymers

**Fig. 6:**
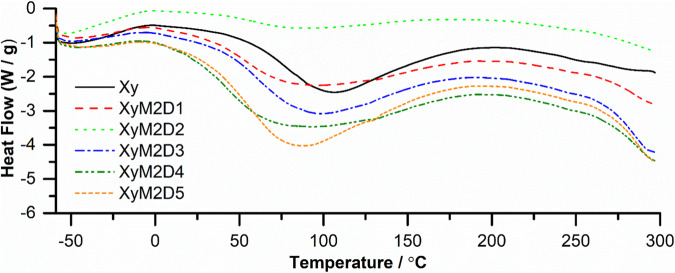
DSC of Xy and Xy-g-PHEAA copolymers

The thermal analyses of Xy and Xy-g-PHEAA synthesized at different doses are shown in Figs. 4–6. TGA was used to study the degradation behavior of Xy compared with that of the grafted derivatives (Fig. 4). The control PS (Xy) showed high thermal stability from approximately 270 °C to approximately 340 °C. Other xyloglucans have shown similar stability in the 300–370 °C range [34]. The chain packing and stiff cellulose backbone of Xy endow this molecule with an unusual thermal stability, often comparable to that of cellulose [35]. The control decomposed in one step, while Xy-g-PHEAA underwent two decomposition steps. It follows that the decomposition steps are the first thermodynamic evidence of grafting.

Additionally, the copolymers showed higher thermal stability than the control. With PHEAA as the grafted polymer, the weight loss began at approximately 270 °C and ended at 400 °C. The first derivative (DTGA) showed that Xy had a Td at 300 °C, and the copolymers exhibited two decomposition peaks at increased temperatures, 330 and 411 °C, confirming PHEAA grafting (Fig. 5).

The DSC plots of Xy and Xy-grafted PHEAA products are shown in Fig. 6. The data on thermodynamic properties are summarized in Table 1. The DSC analysis was carried out without prior treatment (∆Hm_1_(J/g), Tm_1_/(°C), Fig. 6) and after eliminating the thermal history of the PS (∆Hm_2_(J/g), Tm_2_/(°C), ∆H_c2_(J/g), T_c2_/(°C), T_g2_/(°C), Figs. S2–S7, supplementary information).

**Table 1:** Data describing the thermodynamic properties obtained from the thermal analyses

Sample	Td/°C	∆Hm_1_(J/g)	Tm_1_/°C	∆Hm_2_(J/g)	Tm_2_/°C	∆Hc_2_(J/g)	Tc_2_/°C	Tg_2_/°C
**Xy**	324	235.40	104.73	5.74	303.81	18.97	321.35	258.50
**XyM2D1**	330/411	204.15	83.01	30.58	297.97	68.47	326.41	132.32
**XyM2D2**	330/411	161.60	82.55	49.59	299.87	59.47	326.09	157.85
**XyM2D3**	330/411	212.50	94.45	21.71	293.71	27.67	351.15	106.18
**XyM2D4**	330/411	216.50	77.90	28.94	326.74	72.55	326.74	144.34
**XyM2D5**	330/411	248.80	83.20	27.83	328.08	40.83	328.08	129.65

*Td* decomposition temperature, *∆Hm1* melting enthalpy (first heating without previous treatment [FHWPT]), *Tm1* melting temperature (f FHWPT), *∆Hm2* melting enthalpy (thermal history eliminated [THE]), *Tm2* melting temperature (THE), *∆Hc2* enthalpy of crystallization (THE), *Tc2* crystallization temperature (THE), *Tg2* transition temperature (THE)

The first approach allowed us to observe the decreases in ∆Hm_1_ and Tm_1_ for Xy-g-PHEAA relative to those of native Xy. This method showed several endotherms for the polysaccharides directly heated in the DSC instrument (Fig. 6). This decrease was also observed for thiolated Xy and was indicative of PHEAA grafting [36]. The heating was stopped at 300 °C; therefore, the exotherms were not compared initially. Presumably, the changes in melting enthalpies can be attributed to modifications in the degree of crystallinity due to grafting copolymerization [37]. The second approach showed endothermic and exothermic valleys (Figs. S2–S7, supplementary information). The melting temperature (Tm_2_/(°C)) trend coincided with the first method in the 5–15 kGy range. The Tm_2_ of Xy-g-PHEAA increased in relation to Xy after 15 kGy, suggesting possible crosslinking at high doses [38]. Notably, after thermal history elimination, the Xy melting enthalpy (∆Hm_2_) decreased to 5.74 J/g and increased after grafting for all copolymers [8]. The results suggested that after PHEAA grafting, the heat energy necessary to melt the biopolymer was higher for the derivatives than for the unmodified PS [39]. The enthalpy of crystallization (∆Hc_2_/(J/g)) followed the trend as ∆Hm_2_. However, a higher temperature was needed for the crystallization of the grafted hemicellulose than for that of Xy, which is consistent with previous findings [34]. Finally, the shift in the glass transition temperature (Tg_2_/°C) to lower values for grafted molecules indicated a decrease in chain rigidity and less restricted polymer mobility. This outcome was indicative of intermolecular interactions more characteristic of graft polymerization than a crosslinked network [40]. The DSC results corroborated that Xy-g-PHEAA copolymers were formed with improved thermal stability and the least limited structure mobility, which may be helpful in further preparation of scaffolding for STE [41].

### Cytotoxicity and viability assays

**Fig. 7:**
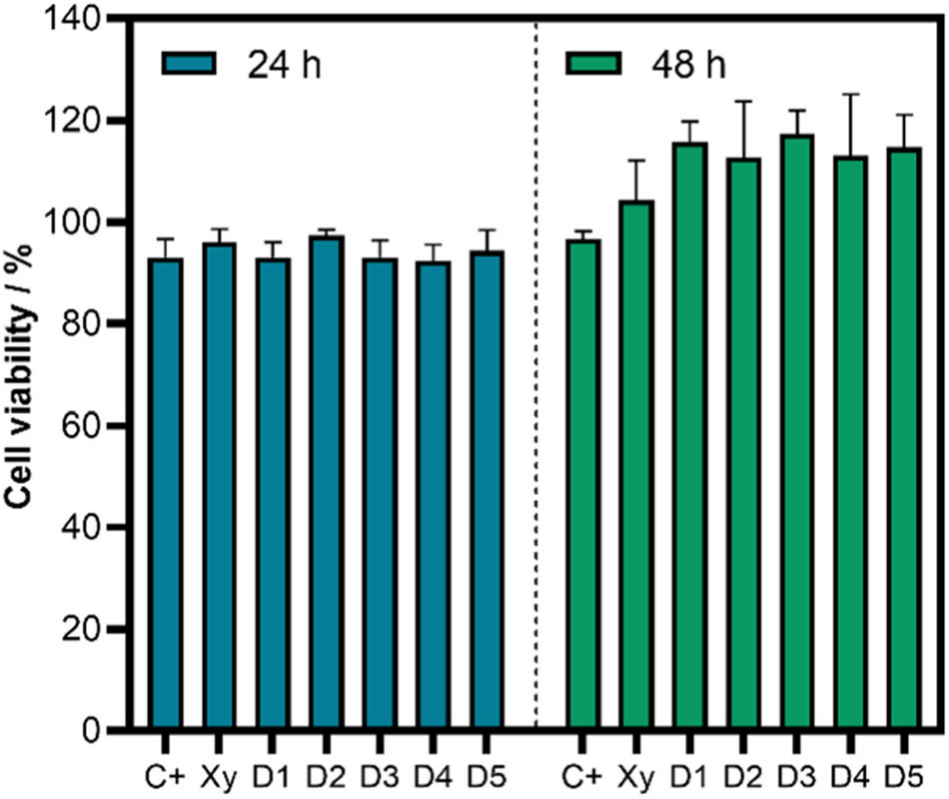
MTT assay for the monolayer (Ctrl+), Xy, and Xy-g-PHEAA after 24 and 48 h of culture. The positive control (Ctrl+) is represented as C+, and the copolymers Xy-g-PHEAA, previously denoted as XyM2D1, XyM2D2, XyM2D3, XyM2D4, and XyM2D5, are labeled as D1, D2, D3, D4, and D5 due to space limitations in the graphs

Figure 7 shows the results of the MTT test for HDFs cocultivated separately with Xy-g-PHEAA and Xy. HDFs cultivated in monolayers were used as the primary control. The metabolic activity of the groups of cells cocultivated with Xy-g-PHEAA at different doses was not significantly different from that of the controls at 24 and 48 h. A trend toward increases in the optical density (OD) with the dose and compared to the controls was observed when comparing the data at 48 h. Fibroblast proliferation was higher at 48 h than at 24 h. The HDF survival rate of over 100% for all samples in the studied periods is consistent with that of biocompatible and nontoxic biomaterials [42].

**Fig. 8:**
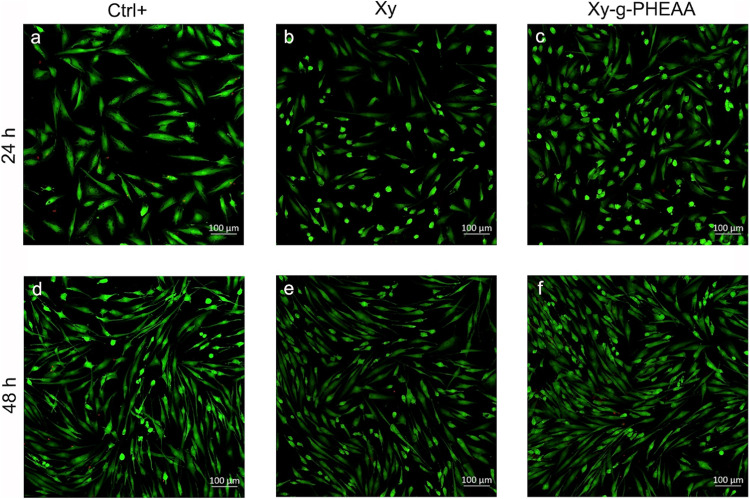
The percentage of live and dead cells for each period was estimated by using a calcein/EthD-1 kit

The calcein/EthD-1 assay was also carried out to complement the MTT results (Fig. 8). From the micrographs, it was observed that most HDFs were alive, with excellent proliferation for Xy and the graft copolymers (representative image of Xy-g-PHEAA). The Live/Dead™ kit did not detect any dead cells when the EthD-1 dye was used within 48 h from the start of the experiment. In previous work, the cytotoxicity of Xy hydrogels was evaluated using L929 cells. The authors observed 100% fibroblast viability after 48 h of incubation, and the concentration ranged from 1 to 100 μg/ml [43]. Moreover, Xy-based hydrogels prepared from the combination of this PS with honey-loaded Gantrez^®^ showed low cytotoxicity toward NHF cells [44]. The increase in hydrophilicity and chemical modifications allowed the formulation of patches for wound care. Furthermore, investigations were previously conducted with extracts of tamarind at different concentrations (1–10 μg/ml) and normal human dermal fibroblasts (NHDFs), and it was concluded that these cells exhibited no cytotoxic effects [23]. In addition, PHEAA has been identified as a hydrogen-bond donor and acceptor and a biocompatible polymer with good bacterial adhesion resistance and capacity to support fibroblast growth in vitro [45].

### Proliferation assays and surface detection

**Fig. 9:**
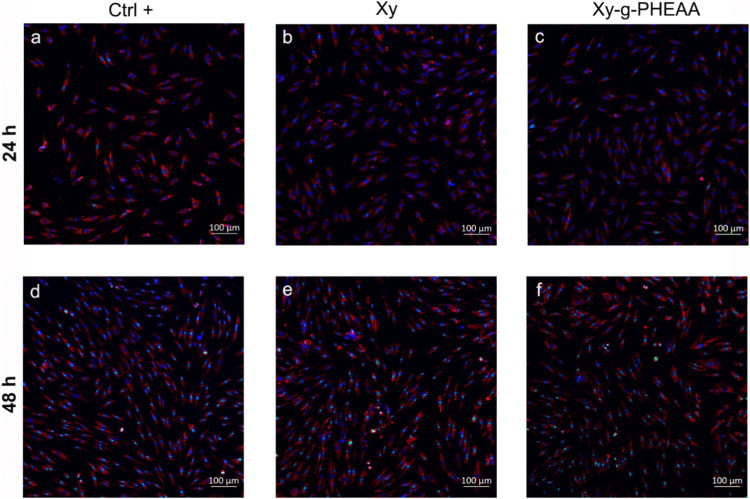
IF staining of HDFs using anti-fibroblast surface protein (1B10) and AF594 (cell membrane-red, nuclei-blue counterstained with Hoechst) and fluorescent labeling of anti-Ki67 and AF488 (green channel in nuclei): **a, d** well plates (control+), **b, e** Xy, and **c, f** representative Xy-g-PHEAA after 24 and 48 h of culturing

Ki67 immunofluorescence staining was performed to assess the role of Xy and Xy-g-PHEAA in HDF proliferation (Fig. 9). The 48-h Ki67 staining results succeeded in showing cell proliferation. A visual inspection of the micrographs revealed a high amount of cell proliferation, with no significant difference in intensity between the grafted Xy and the controls (Ctrl+, Xy). Additionally, the variation in the number of Ki67-positive HDFs per field was unimportant [46–49]. Furthermore, 48 h 1B10 staining revealed that cells cocultivated with Xy and Xy-g-PHEAA recognized, with the same specificity of fibroblasts as the culture plate cells. This finding indicated that the fibroblast phenotype was not affected.

### HDFs migration experiment

**Fig. 10:**
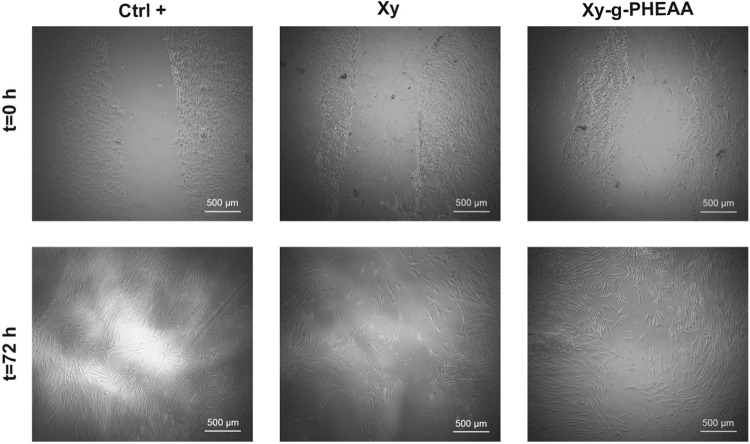
A representative migration experiment from a confluent monolayer (Ctrl+), monolayer +Xy, and monolayer + Xy-g-PHEAA of HDFs into the scratch area was conducted for 72 h. The bar code for the experiment micrographs = 500 μm

HDFs (monolayer, Ctrl+), HDFs (monolayer) + Xy, and HDFs (monolayer) + Xy-g-PHEAA, were used in the migration experiments (Fig. 10). After 72 h, HDFs cocultivated with Xy-g-PHEAA exhibited a smaller remaining closure area than the sample cocultivated with Xy, clearly demonstrating accelerated migration. These findings support the idea that the introduction of amino groups through gamma radiation-induced graft polymerization of HEAA onto Xy leads to the creation of new molecules with improved properties for biomedical use. In this case, it results in a greater stimulation of migration, which is a crucial process in tissue regeneration. Dutta et al. reviewed the use of Xy hydrogel in the neural cell migration model of a scaffold implanted in mice Parkinsonian brain [50]. According to Deters et al., Xy enhanced fibroblast and keratinocyte proliferation, accelerated fibroblast migration and intracellular enzyme activity following endosomal uptake, and supported the cell cycle [23]. Additionally, special wound healing patches made of honey loaded Gantrez^®^/Xy hydrogel were prepared, motivated by the high efficiency of Xy in stimulating fibroblast migration [44]. Certainly, the ungrafted Xy exhibits remarkable properties in stimulating fibroblast migration, establishing a solid foundation. The findings for Xy-g-PHEAA signify a significant advancement, opening avenues for the development of novel formulations tailored for dressings and patches in the treatment of burn induced wounds.

## Conclusion

In summary, Xy was successfully grafted with PHEAA by radical polymerization using gamma radiation as an initiator. The analysis of the proposed reaction mechanism indicated that the grafting reaction necessarily occurred on Xy hydroxyl groups. The chemical changes that verified the introduction of PHEAA moieties onto Xy were observed by spectroscopic and thermal studies. The graft copolymer Xy-g-PHEAA showed no adverse impact on HDF cytotoxicity, viability, or proliferation. The Xy subjected to grafting exhibited migration capabilities superior to those of the pristine polymer. The biocompatibility of the grafted Xy suggested that the new molecules had good properties for application in STE. An interactive molecule was designed with more capacity for forming hydrogen bonds and polymer networks than pristine xyloglucan. However, the work was limited to experiments with HDFs cocultivated with the copolymer. Further preparation of graft copolymer hydrogels is needed to produce scaffolds with adequate mechanical properties and resistance to bacterial adhesion for clinical practice.

## supplementary information

supplementary information
