# Supplementary material for: Preparation of xyloglucan-grafted poly(N-hydroxyethyl acrylamide) copolymer by free-radical polymerization for in vitro evaluation of human dermal fibroblasts
Source: J Mater Sci Mater Med. 2024 Mar 25;35(1):20. doi: 10.1007/s10856-024-06783-1 (PMC10963570; doi:10.1007/s10856-024-06783-1)
Supplement: Supplementary file 1 — supplementary information [file 10856_2024_6783_MOESM1_ESM.docx]

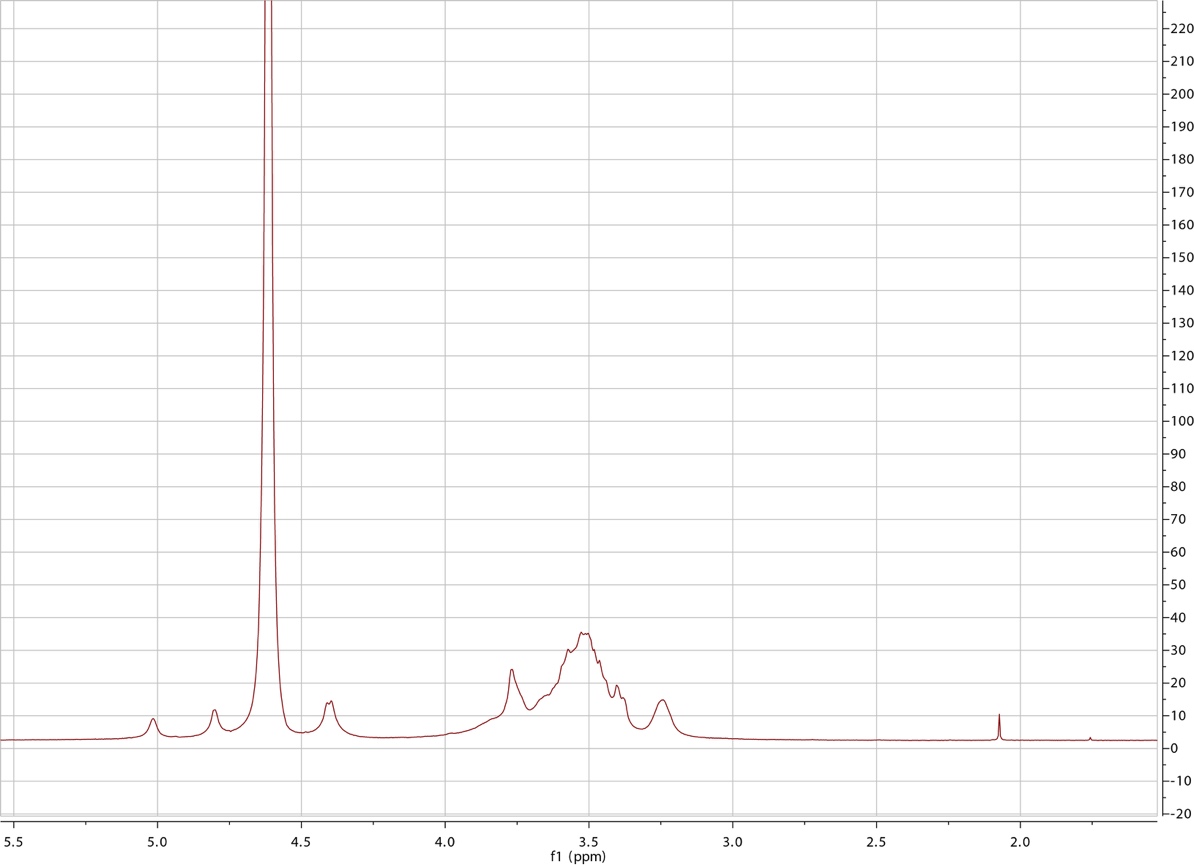


**Fig. S1** ^1^H-NMR of Xy


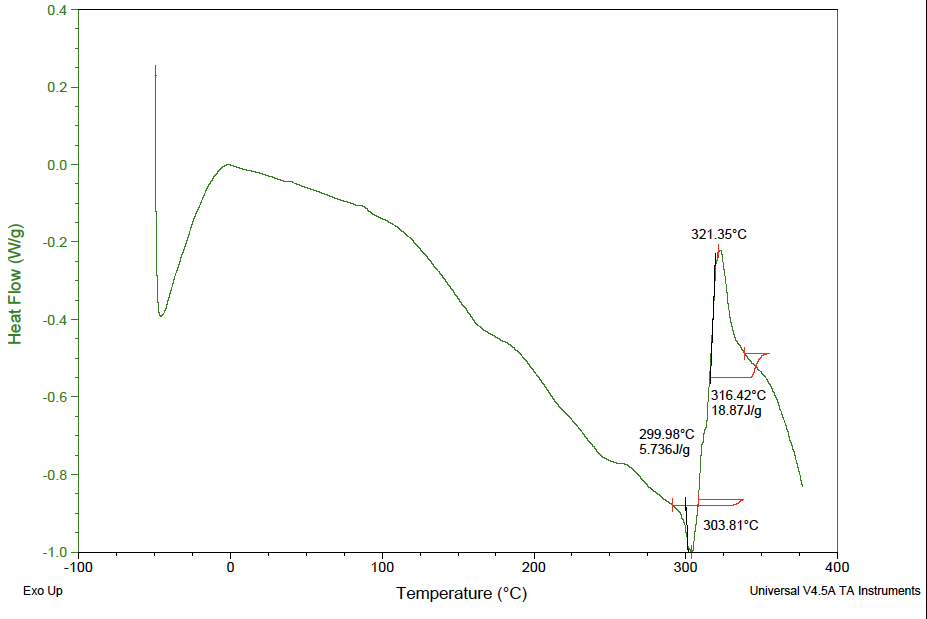


**Fig. S2**. DSC of Xy


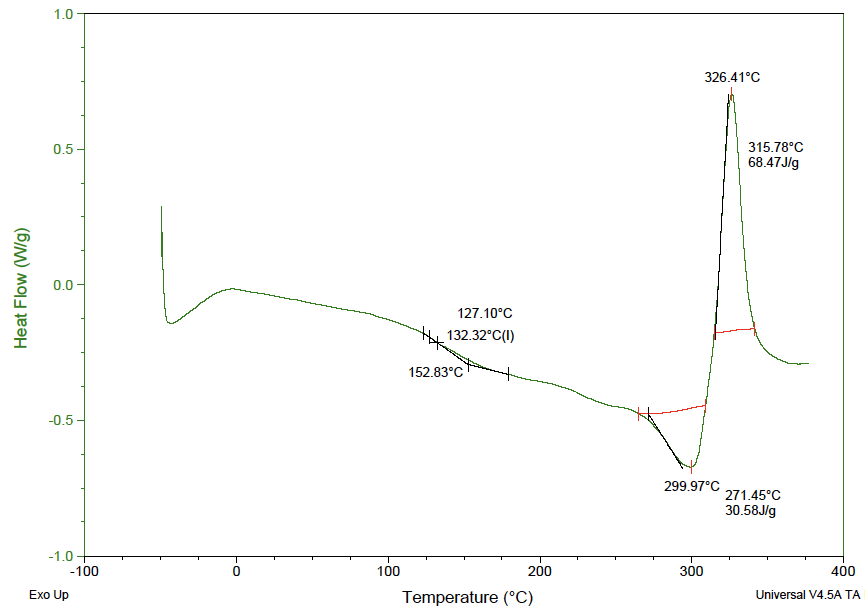


**Fig. S3.** DSC of Xy-g-PHEAA (XyM2D1)


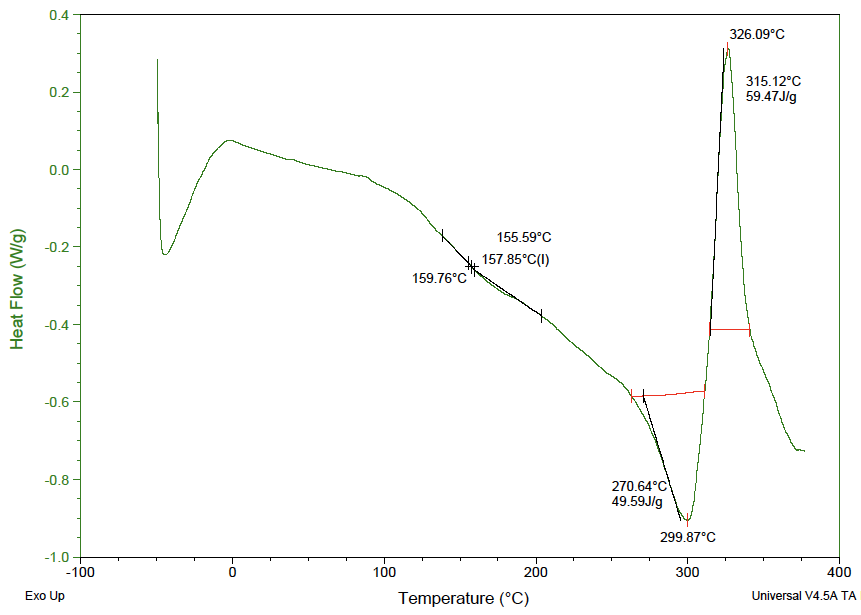


**Fig. S4.** DSC of Xy-g-PHEAA (XyM2D2)


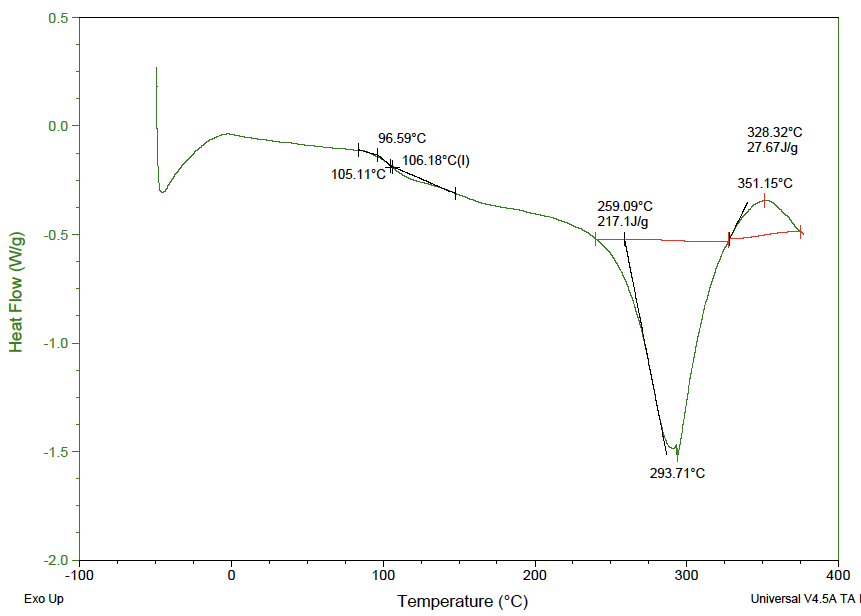


**Fig. S5**. DSC of Xy-g-PHEAA (XyM2D3)


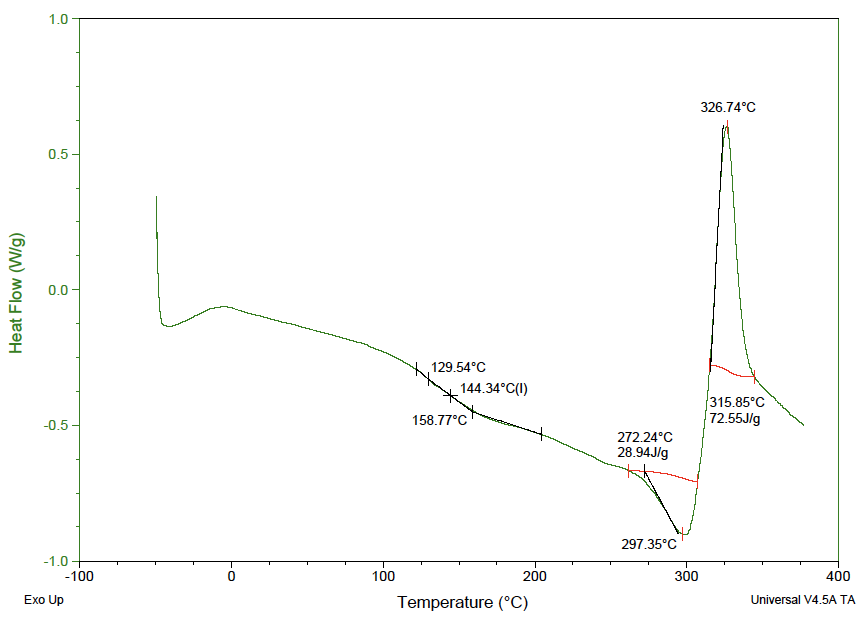


**Fig. S6.** DSC of Xy-g-PHEAA (XyM2D4)


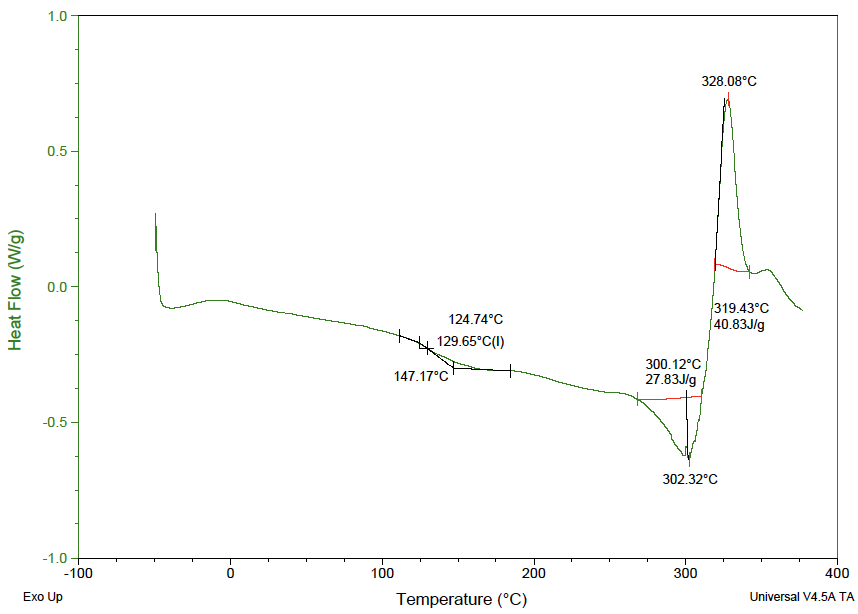


**Fig. S7.** DSC of Xy-g-PHEAA (XyM2D5)
